# Analysis of critical states based on acoustic emission signals during progressive failure of wood

**DOI:** 10.1371/journal.pone.0302528

**Published:** 2024-05-16

**Authors:** Xiaoyan Jia, Junqiu LI, Qinghui Zhang, Meilin Zhang, Yiting Jin, Yang Ding

**Affiliations:** Department of Big Data and Intelligent Engineering, Southwest Forestry University, Kunming, Yunnan, China; University of Vigo, SPAIN

## Abstract

The analysis of critical states during fracture of wood materials is crucial for wood building safety monitoring, wood processing, etc. In this paper, beech and camphor pine are selected as the research objects, and the acoustic emission signals during the fracture process of the specimens are analyzed by three-point bending load experiments. On the one hand, the critical state interval of a complex acoustic emission signal system is determined by selecting characteristic parameters in the natural time domain. On the other hand, an improved method of b_value analysis in the natural time domain is proposed based on the characteristics of the acoustic emission signal. The K-value, which represents the beginning of the critical state of a complex acoustic emission signal system, is further defined by the improved method of b_value in the natural time domain. For beech, the analysis of critical state time based on characteristic parameters can predict the “collapse” time 8.01 s in advance, while for camphor pines, 3.74 s in advance. K-value can be analyzed at least 3 s in advance of the system “crash” time for beech and 4 s in advance of the system “crash” time for camphor pine. The results show that compared with traditional time-domain acoustic emission signal analysis, natural time-domain acoustic emission signal analysis can discover more available feature information to characterize the state of the signal. Both the characteristic parameters and Natural_Time_b_value analysis in the natural time domain can effectively characterize the time when the complex acoustic emission signal system enters the critical state. Critical state analysis can provide new ideas for wood health monitoring and complex signal processing, etc.

## 1 Introduction

Wood is a green, environmentally friendly and widely distributed bio-renewable material [1] that has been used as a basic building material in human society for a long time because it is easily available and has good support properties. At present, the existing ancient buildings in addition to some special stone buildings are almost all wooden buildings, due to the war, lack of routine maintenance and natural disasters such as earthquakes, typhoons and other natural disasters will cause different degrees of damage to the wooden building materials [2]. Therefore, it is necessary to monitor the state of ancient buildings through modern technological means, and if the state of wooden materials can be accurately judged, then it will be very meaningful for the health monitoring of ancient buildings as well as routine maintenance. In the 21st century, the development of miniaturization and wireless sensing technology has led to the rapid development of health monitoring technology, which is widely used in bridges, machinery and equipment, and large-scale ancient wooden buildings, etc [3]. The combination of computer technology and sensing technology has brought new ideas for structural monitoring. The combination of computer technology and sensing technology has brought new ideas for structural monitoring. Ye X-W et al. [4] proposed a method to detect wood features and automatically classify defects in wood images collected using a laser scanner by means of a deep convolutional neural network with an overall accuracy of 99.13%. Peng X et al. [5] proposed an image recognition technique based on UAV-based R-FCN network combined with Haar-AdaBoost crack recognition method for bridge crack identification and width quantification by learning high-level features, shape features and grayscale features of cracks, and ultimately the real bridge crack width quantification accuracy reaches more than 90%. Oh J-K et al. [6] investigated a machine vision system for automatic crack detection.The machine vision system takes images as inputs and produces other types of outputs such as crack lengths, crack widths, and contour sketches of the bridge condition.The use of the machine vision system ensures the accuracy of the crack assessment and provides a wide range of information for the maintenance of bridges through the results of the bridge inspection. The studies on structure monitoring by machine vision have all achieved relatively good results, but there are high requirements for acquisition equipment during the image acquisition process and may be affected by factors such as light and line of sight leading to high monitoring costs for obtaining usable images. A more fatal disadvantage of machine vision monitoring is the inability to obtain an effective image for analysis when the monitoring object is in a position where the camera equipment is unable to take a picture, e.g., the crisscrossing of wooden parts in mortise-and-tenon joinery, which is often used in wooden buildings, which leads to the failure of machine vision monitoring. On the other hand, defects detected by visual monitoring have caused serious irreversible damage to the structure, so it is very important to choose a non-destructive monitoring method that can easily access the monitoring data and thus be able to provide an early warning before serious damage occurs.

When a material undergoes inelastic deformation, some of the energy released will propagate through the material in the form of elastic waves, which is known as AE (acoustic emission, AE) [7]. In recent years, advanced non-destructive testing methods such as AE techniques are particularly suitable for fatigue damage monitoring, mainly because of their high sensitivity to inelastic material changes such as microcracks. Related studies [8–10] have shown that the internal structure of wood is complex, so the path of AE signal conduction in wood is very complicated, and the collected AE signal is characterized by non-smoothness, non-linearity and complexity. Aldahdooh MAA et al. [11] classified the type of cracks (flexural or shear) in several types of four-point bending RC beams based on the fracture mechanism of the RC beams and the characterization of the AE signals, and the final results obtained were in agreement with the results of visual observations based on the crack patterns. Li M et al. [12] investigated the propagation characteristics of AE signals along the wood grain direction based on discrete wavelet analysis and established an energy attenuation model for AE signals, which showed that the energy of AE signals decayed exponentially with the propagation distance when the surface transverse wave and the internal longitudinal wave propagated through the wood. Nasir V et al. [13] systematically reviewed the AE method and its application to the wood and timber industry and discussed the effect of wood properties on the velocity and energy attenuation of the AE signal. Diakhate M et al. [14] compared cluster analysis of AE data with numerical modeling for the assessment of crack length evolution, and the results demonstrated a good correlation between the AE results and numerical predictions. The above studies have demonstrated that AE technology has been widely used to monitor the structural integrity, mechanical behavior, and damage detection of wood materials, so AE signals are an effective tool for studying the internal state of wood. Our group previously Zhang M et al. [15] proposed a signal classification method for wood AE signals regarding the internal damage state of wood, but the results made it difficult to distinguish between microcracked AE signals and deformed AE signals. This is due to the fact that the features that can accurately identify the AE signals of microcracks were not selected in the establishment of the classification model. The microcrack stage in the process of wood fracture is also the stage of the critical state of wood, so the investigation of the characteristic parameters that can indicate that the wood enters into the interval of the critical state is very meaningful for the classification of the internal state of wood. According to the published studies, most of the current studies are only based on the statistical characteristics of the AE signals to classify the internal damage types or to study the propagation characteristics, compared to the analysis of the critical state of wood, which is almost non-existent. However, the critical state is a very important stage for the overall wood breakage process. An uncontrollable "system collapse" event occurs when the system is subjected to external disturbances that reach a critical state in a very short period of time, and when this event occurs it will result in a devastating blow to the system as a whole. The study of the critical state of the timber fracture process can effectively capture the occurrence of the critical state, which can lead to early warning and ultimately prevent the occurrence of such a catastrophic collapse event. Further investigation will continue in this study for the AE signal during wood fracture to determine the critical state interval of wood. The performance of timber structures in construction has a direct impact on the safety and stability of the building [16], and understanding the critical state of wood under different moisture and temperature conditions can help to design longer-lasting, more reliable timber structures that will remain stable in a variety of environments. The study of the critical state of wood in forestry helps to promote the sustainable use of wood, and by gaining a deeper understanding of the properties of wood, forest resources can be better managed [17], the risk of over-harvesting can be reduced, and the sustainable production and use of wood can be promoted. Overall, the study of the critical state of wood not only contributes to improved engineering applications of wood, but also has far-reaching implications for environmental sustainability and ecological balance, providing a basis for effective management and utilization of wood resources.

Wood is mainly composed of cellulose, hemicellulose, and lignin, and the proportions of these major components vary between and within species, as well as between and within individual trees, and it is an anisotropic material due to its unstable composition [18]. Therefore, the AE signals collected by the sensors are difficult to capture effective feature information in the traditional time domain, and can only roughly determine the region where the fracture occurs based on its time-amplitude. So it needs to be converted to different scales for further analysis to obtain more effective information. The natural time domain was introduced in 2001 as a method to analyze time series generated by complex systems. And the analysis under natural time can break through the limitations in traditional time by focusing not only on amplitude fluctuations in continuous time, but on the sequence of events and occurrences between events, emphasizing the importance of the events and the events themselves[19–21]. In recent years, the analysis of natural time domain for signals involved in natural disasters, structural monitoring, medicine and other fields have achieved good results, proving that new dynamic features hidden behind time series in complex systems can be analyzed by analyzing them in the natural time domain. Triantis D et al. [22] by utilizing experimental data provided by two sensing techniques, acoustic emission and pressure-stimulated current, Lili found that both techniques provide significantly different critical indices by comparative analysis of acoustic and electrical activity in the natural time domain. Varotsos PA et al. [23] observed earthquake scaling laws indicate the existence of phenomena closely associated with the proximity of the system to a critical point. Varotsos PA et al. [24] discussed that the parameters under natural time analysis are such that it is possible to extract as much information as possible from complex system time series for earthquake prediction. Triantis D et al. [25] explored the possibility of detecting indices through a discussion of parameter-specific variations in the natural time domain, showing that the evolution of the average rate of change of the cumulative counts in the natural time domain of impending catastrophe provides an indicator of whether or not the applied load is approaching the value at which the specimen enters the critical state of impending fracture. Triantis D et al. [26] analyzed the acoustic emissivity based on the natural time concept and analyzes the experimental data of cementitious materials under three-point bending, concluding that if the acoustic activity is described with the help of F-functions in the natural time domain, its evolution is governed by a power law independent of the geometrical details and of the type of loading scheme, and that the validity of this law seems to provide an interesting pre-failure indicator. Loukidis A et al. [27] employed a natural time concept to describe the temporal evolution of the F function, whose value increases gradually with fluctuations of different intensities; however, a power law seems to systematically govern the response of the loaded structure as fracture approaches, which provides a useful pre-fracture signal. Baldoumas G et al. [28] distinguished between congestive heart failure patients and healthy patients by applying natural time analysis, and the results of their study showed that analysis in the natural time domain can successfully distinguish between the two. Arroyo-Solórzano M et al. [29] proposed a new estimate of the Gutenberg-Richter relationship by combining seismic data from the National Seismic Network and several Central American catalogs for the years 1522 to 2020, and explored the link between b_value and seismic spatial-temporal relationships by calculating the completeness magnitude and applying a temporal window declustering approach to a seismic catalog containing about 122,000 earthquakes. Carpinteri A et al. [30] investigated the continuous damage process present in the masonry walls of the Assinelli Tower in Bologna using the AE technique, deriving from the AE time series the trends of the two evolutionary parameters b_value and the natural time variance, in order to determine the degree of proximity of the monitored structural units to the critical state associated with the occurrence of earthquakes. Lei X et al. [31] quantitatively modeled the AE activity based on the b_value of the magnitude-frequency relation, the self-excitation intensity of the AE time series, and the fractal dimension of the source. And the analysis revealed three long-term phases of the AE activity associated with the generation of non-homogeneous fault damage, and each of the phases can be clearly identified based on the above parameters. The above study justifies the use of natural time domain analysis in the problem of determining the critical state. Since the appearance of high magnitude in seismic statistics represents the occurrence of a "catastrophic" event, and the appearance of high amplitude in the process of wood fracture represents the occurrence of irreversible "damage" to the wood, there is a certain degree of similarity between the AE signals in the process of wood fracture and the seismic signals. So it is feasible to use the b_value to analyze the damage state.

In this study, we focus on how to determine the critical state intervals of AE signals in the natural time domain, using beech trees—beech, family Elmaceae, and pine trees—camphor pine, family Pinaceae, as experimental materials. Sphagnum pine is characterized by straight grain, light and soft, medium toughness and medium dry shrinkage, and beech is characterized by straight grain, heavy and hard, high toughness and high dry shrinkage. Arborvitae is often used as a building material because of its fine structure, tall and upright, and the characteristics of a successful trunk, and it is widely used in ancient architecture [32]. The acquired AE signals inevitably contain a lot of noise due to the influence of the environment and acquisition equipment, etc. Firstly, the noise reduction of the AE signals is carried out by the blind deconvolution algorithm, and then the interval range of the critical state of the complex AE system is determined by analyzing the AE signals in the traditional time domain, the natural time domain, and the improved b_value.

## 2 Materials and methods

### 2.1 Experimental setup

**Fig 1:**
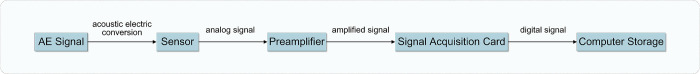
AE signal acquisition process diagram.

Fig 1 illustrates the AE signal acquisition process adopted in this study. Initially, the sensor captures the AE signal resulting from the surface pressure exerted on the specimen, which is then transformed into an analog signal through acoustic-electrical conversion. Subsequently, the analog signal undergoes amplification via a preamplifier, yielding the amplified signal. Finally, the amplified signal is digitized by a data acquisition card and stored in the computer as a digital signal. To facilitate subsequent analyses, the AE signals are segregated using LabVIEW software, thereby saving the individual AE signals received by the sensors into separate text files.

The experimental equipment models for this study are listed below:

1. NI USB-6366 high-speed acquisition card: This model boasts a maximum sampling frequency of 2 MHz. It enables the upload of AE signals to the computer via the high-speed acquisition card’s USB interface.
2. UTM5105 Universal Mechanical Testing Machine: This model has a power of 1.5 kW and a maximum test force of 100 kN.
3. SR 150 N Single-Ended Resonant AE Sensor: This model is used at temperatures ranging from -20°C to 80°C, with a signal bandwidth of 25–200 kHz, and is characterized by high sensitivity and high temperature resistance.
4. 40 dB gain preamplifier: This amplifier has a maximum sampling frequency of 2 MHz, an output voltage range of ±5 V, and built-in noise reduction to facilitate signal transmission.
5. LabVIEW software-based signal acquisition system: This acquisition system realizes the extraction, separation and storage of AE signals received by the data acquisition card.

### 2.2 Experimental procedure

This study employed a three-point bending load experiment to apply transverse constant displacement to specimens at a rate of 1 mm/min. Beech and camphor pine wood without surface defects and with dimensions of 800 mm (length) × 60 mm (width) × 30 mm (thickness) were selected as the subjects for the study, and the specimens were air-dried and their moisture content was stabilized at about 11%. It is worth noting that wood, as a unique anisotropic biological material, is greatly influenced by its moisture content, which can impact its physical properties. The choice of an 11% moisture content value was based on considering the typical moisture content range of wooden components in ancient architecture, which generally falls between 6% and less than 28% [33].

**Fig 2:**
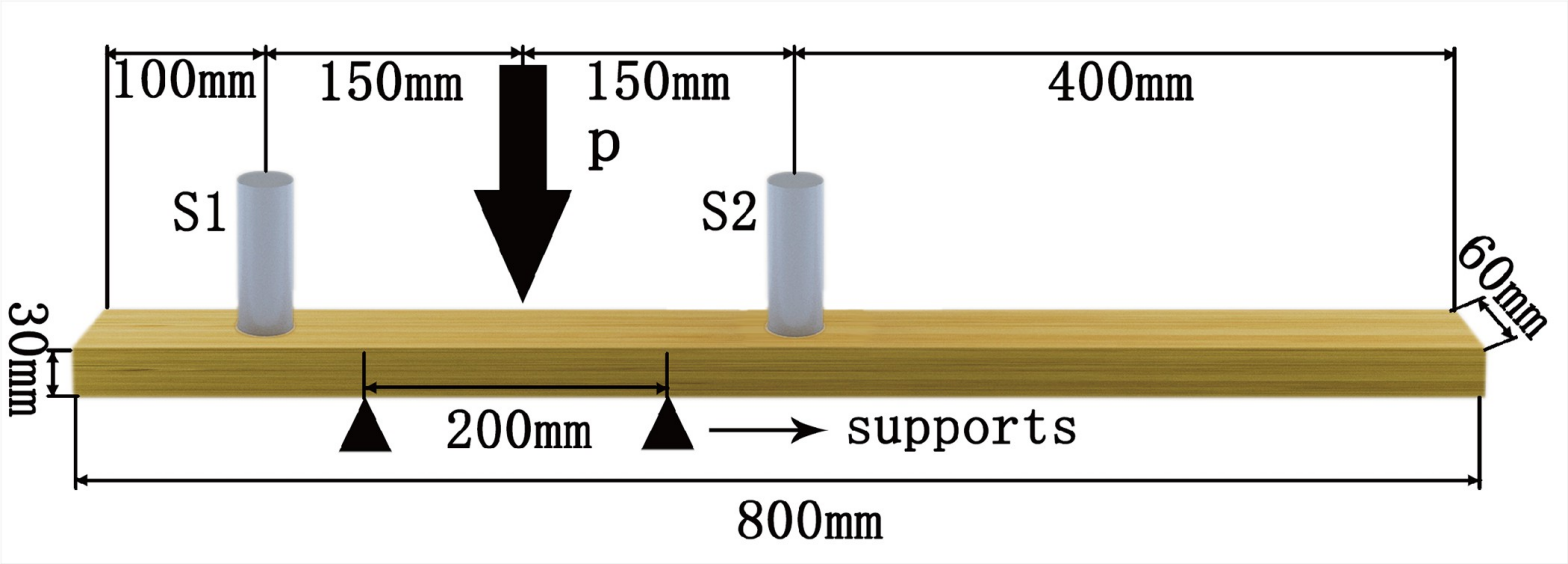
Experimental setup diagram.

The experimental setup is shown in Fig 2 and consists of two support points, two sensors (S1 and S2) and a pressurization point (P). The distance between the two support points is 200 mm, the distance of P from each of the two support points is 100 mm, the distance of S1 and S2 from P is 150 mm, the distance of S2 from the right edge of the wood sample is 400 mm, and the distance of S1 from the left edge of the wood sample is 100 mm. To ensure precise signal collection, a coupling agent in the form of silica gel is applied to the sensor [34]. It is important to note that the penetration of the coupling medium into the wood samples may introduce experimental errors. Therefore, efforts should be made to minimize the impact of the coupling agent on the wood specimens by reducing the experiment’s duration and carefully controlling the amount of coupling agent used.

### 2.3 Blind deconvolution algorithm

The AE signal data in the experiment is obtained after processing by the sensor, preamplifier and data acquisition card. Although the noise interference is minimized during the experiment, the original AE signal is still interfered by factors such as propagation medium, machine noise, and the degree of sensor coupling during AE signal propagation, and these uncontrollable influences result in distortion of the signal. After simplifying the propagation path the acquisition process can be expressed as Eq (1), *X(t)* is the original AE signal, *Z(t)* is the propagation path system, and *Y(t)* is the stored AE signal. In practice it is expected that *Y(t)* and *X(t)* will be maximally similar. If both *X(t)* and *Z(t)* are unknown and the real original AE signal can only be estimated from the measured signal *Y(t)*, then there is some blindness in solving the source signal *X(t)*, which is called blind inverse convolution. The goal of blind deconvolution is to design a filter h(t) that takes the acquired AE signal *Y(t)* and processes it through t to obtain a signal similar to the original AE signal [35].


Y(t)=X(t)*Z(t) (1)

The MED (Minimum Entropy Deconvolution, MED) algorithm is a blind deconvolution technique widely used in fault detection [36–38]. Initially proposed by Wiggins [39] for seismic recording, MED has found applicability in various domains. In typical signal processing scenarios, noise reduction algorithms are selected based on a priori knowledge of the noise signal or the frequency distribution characteristics of the original signal. However, due to the unique properties of the experimental material in this study, such knowledge is unavailable, making it challenging to reconstruct waveform signals based solely on frequency domain differences between the effective and noise signals. Additionally, the special internal structure of wood lacks a well-established mathematical-physical theoretical foundation for propagation paths, limiting the availability of exploitable statistical values for noise reduction. Previous studies have indicated that early wood cell wall peeling exhibits low AE energy while cell wall tearing produces high AE energy [40]. In this study, noise interference originates from environmental factors, propagation paths, equipment, and other minor sources, while the collected AE signals represent acoustic signals of fiber breakage within the wood. Consequently, the amplitude of the effective AE signals significantly exceeds that of the noise signals. Leveraging the inherent advantage of not requiring prior knowledge, the MED algorithm is employed for noise reduction in this study. The kurtosis value, a numerical statistic reflecting waveform distribution characteristics, indicates the proportion of shock components in the signal. By selecting a finite-length filter h to approximate the extraction of high kurtosis signal components while minimizing low kurtosis signal components and noise, the MED algorithm achieves noise reduction. In Eq (2), X represents the AE signal acquired by the sensor, and Y denotes the AE signal processed using the MED algorithm.


Y=h*X (2)

The lengths of X and Y are N and the length of h is L, where Y, X and h are described as shown below:

$$Y=[\begin{array}{c} Y_{1}\\ \vdots \\ Y_{N} \end{array}]X=[\begin{array}{c} X_{1}\\ \vdots \\ X_{N} \end{array}|h=[\begin{array}{c} h_{1}\\ \vdots \\ h_{L} \end{array}]$$

Describe Eq (2) in matrix form as shown in Eq (3):

$$Y=X_{0}^{T}h$$ (3)

Where *X*_**0**_ is an *N* × *N* matrix described as follows:

a
$$X_{0}=[\begin{array}{cccccc} X_{1} & X_{2} & X_{3} & \cdots & \cdots & X_{N}\\ 0 & X_{1} & X_{2} & \cdots & \cdots & X_{N-1}\\ 0 & 0 & X_{1} & \cdots & \cdots & X_{N-2}\\ \vdots & \vdots & \vdots & \ddots & \cdots & \vdots \\ 0 & 0 & 0 & \cdots & \cdots & X_{N-L+1} \end{array}]$$

Assuming that the mean value of Y is 0, the kurtosis maximization problem is described as shown in Eq (4):

$$\max\_kurtosis=max\frac{\sum_{n=1}^{N}Y_{n}^{2}}{{\left(\sum_{n=1}^{N}Y_{n}^{2}\right)}^{2}}$$ (4)

The suitable filter h is solved iteratively and the formula for solving h is described as shown in Eq (5):

$$h=\frac{\sum_{n=1}^{N}Y_{n}^{2}}{\sum_{n=1}^{N}Y_{n}^{4}}\left(X_{0}X_{0}^{T}\right)X_{0}{\left[Y_{1}^{3}Y_{2}^{3}Y_{3}^{3}\cdots Y_{N}^{3}\right]}^{T}$$ (5)

The specific calculation steps for MED are as follows:

1. Assuming that the initial filter is the center pulse, $h={\left[0,0,\cdots ,1,\cdots ,0,0\right]}^{T}$.
2. Calculate the $X_{0}、X_{0}X_{0}^{T}$ and Y of the input signal.
3. The new filter coefficients are solved for by Eq (5).
4. Until the output of Y calculated by Eq (3) when the iteration stops.

### 2.4 Natural time domain analysis

It is time, not space, that poses the greatest challenge to science; the traditional model of time is a one-dimensional continuum of real numbers, but this continuum is not derived from fundamental principles [20]. By shifting the focus from the intervals between events to the sequence of events themselves, the analysis of complex signal systems in the natural time domain has the potential to overcome the limitations of the traditional time domain for signals. This approach enables the discovery of additional feature information to effectively characterize the state of signals.

#### 2.4.1 Basic definition

**Fig 3:**
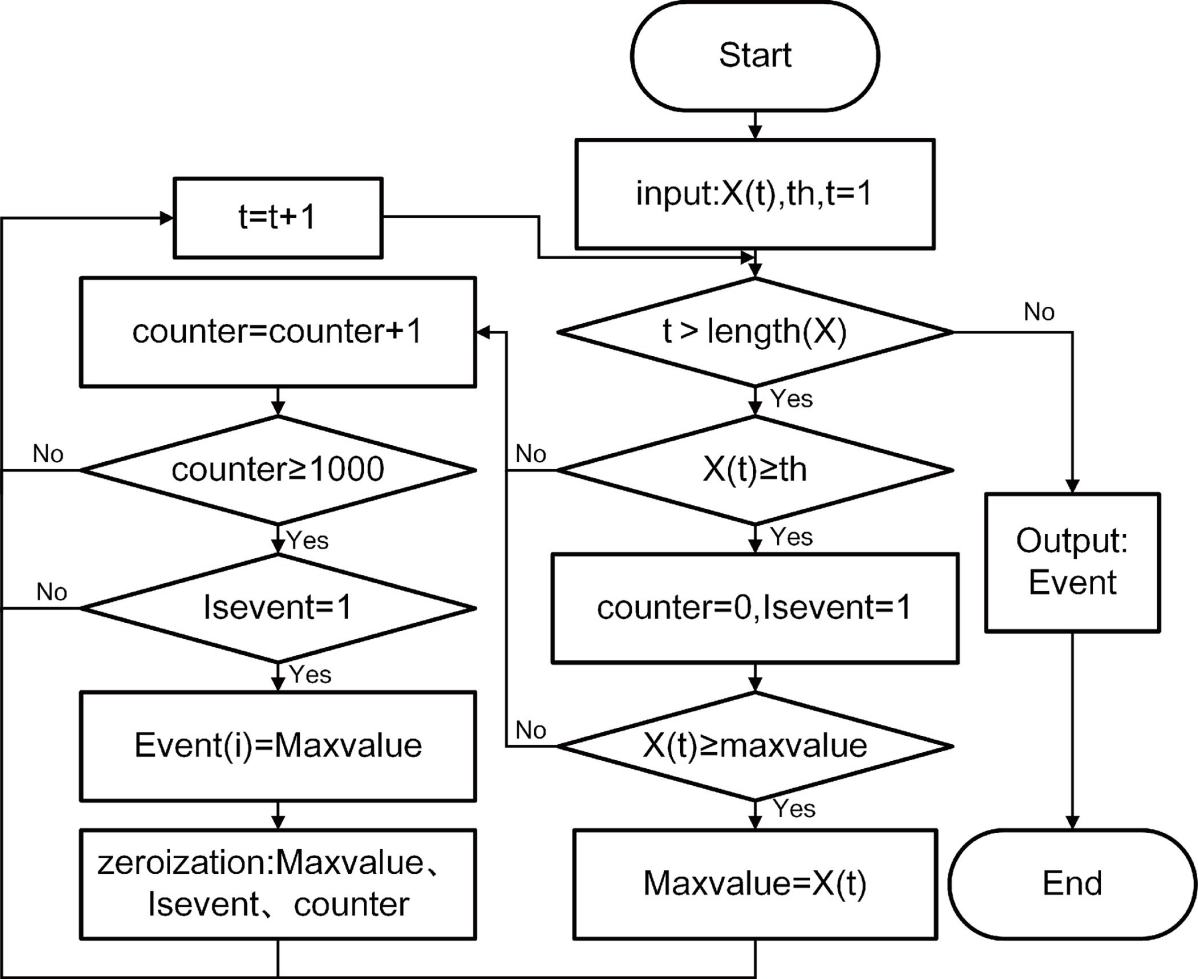
Flowchart for defining events in the natural time domain.


In the natural time domain, when a segment of a signal contains *N* events, *k*th event can be defined using Eq (6), while the normalized energy is specified in Eq (7), and *Q*_*k*_ is energy emitted during an event in natural time. Unlike the traditional time domain, where all AE signals under a single envelope are counted as separate events, the natural time domain definition considers the signal that best represents the occurrence of an AE event under a single envelope as a single event. Consequently, there is only one event per envelope. Fig 3 illustrates the event definition flowchart, which cyclically determines the value of each AE signal and ultimately records the events defined in the natural time domain in the Event variable. A segment of a signal is characterized by $\sum_{k=1}^{M}\chi (k)=1$ if the segment contains M events.

$$\chi (k)=\frac{k}{N}$$ (6)

$$p_{k}=\frac{Q_{k}}{\sum_{n=1}^{N}Q_{n}}$$ (7)

The variance *χ*(*k*), denoted as *κ*1 and calculated using Eq (8), remains unaffected by the time reversal operator $\tilde{T}$:

$$\kappa 1=\langle \chi^{2}\rangle -{\langle \chi \rangle }^{2}=\sum_{k=1}^{N}p_{k}{\left(\frac{k}{N}\right)}^{2}-{\left(\sum_{k=1}^{N}\frac{k}{N}p_{k}\right)}^{2}$$ (8)

The entropy *S* of *χ*(*k*) is calculated according to Eq (9). In the natural time domain, the defined entropy represents the dynamic entropy, capturing the system’s dynamic characteristics as opposed to the static entropy defined in the traditional time domain.


$$S=\langle \chi \ln(\chi )\rangle -\langle \chi \rangle \ln\langle \chi \rangle =\sum_{k=1}^{N}p_{k}\chi (k)\ln(\chi (k))-(\sum_{k=1}^{N}p_{k}\chi (k))\ln(\sum_{k=1}^{N}p_{k}\chi (k))$$ (9)

For a time series with *N* events, the effect of the time reversal operator $\tilde{T}$ on *p*_*k*_ is given by Eq (10) and the effect of the time reversal operator $\tilde{T}$ on *χ*(*k*) is given by Eq (11). The time-reversal entropy *RS* of χ(k) is calculated using the time-reversed $\tilde{T}p_{k}$ and $\tilde{T}\chi (k)$.


$$\tilde{T}p_{k}=p_{N-k+1}$$ (10)

$$\tilde{T}\chi (k)=\chi (N-k+1)=\frac{N-k+1}{N}$$ (11)

The feature parameter map can be obtained after updating *RS*,*S* and *κ*1 several times in the natural time domain by increasing the value of the total number of events *N* each time. The system enters a critical state when the following two conditions are met [19, 41–44]:

1. *κ*1 has a tendency to move from above to below and the value is around 0.07.
2. Both S and RS are smaller than 0.0966.

#### 2.4.2 Natural_Time_b_value

**Fig 4:**
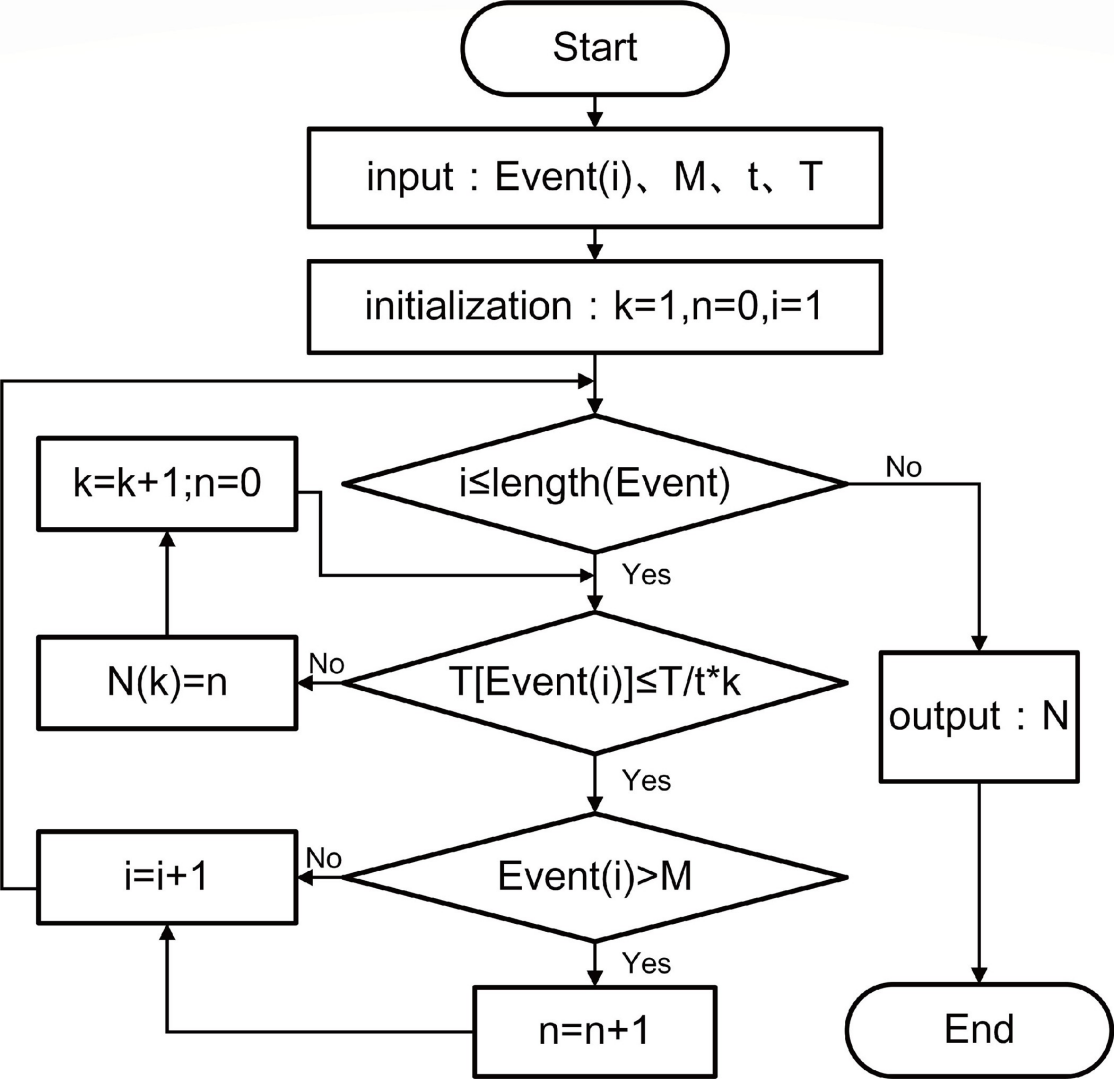
Flowchart for calculating the cumulative value of valid events.


The magnitude-frequency relationship log*N* = *a*−*bM* is a fundamental law in statistical seismology [45]. Here, *M* represents the magnitude of an earthquake, *N* denotes the number of earthquakes equal to or greater than magnitude *M* within a specific time period, and the intercept a depends on the study area and time window. The power-law relationship between the number of events *N* above a given amplitude *M*, derived from AE amplitude distribution data, is commonly known as the Gutenberg-Richter relationship [46] and is expressed in Eq (12). In seismic statistics, the occurrence of high magnitudes corresponds to "catastrophic" events, while high amplitudes in the AE signals indicate irreversible "damage" in the timber rupture process. Due to the similarity between AE and seismic signals, the Gutenberg-Richter relationship is applied to analyze AE signals. A novel method called *NT_b_value*, based on redefined events in the natural time domain, is proposed for calculating the *b-value*. Eq (13) illustrates the calculation method for Natural_Time_b_value (*NT_b_value*). *N* represents the number of events larger than a specified amplitude *M* within a defined range, and the calculation process of *N* is depicted in Fig 4. A higher *NT_b_value* indicates the occurrence of numerous small-amplitude AE events in the specimen, while a lower value suggests the presence of numerous high-amplitude AE events. A significant drop in the *NT_b_value* indicates the imminent instability of the AE system, signifying the entry of the AE signaling system into a critical state referred to as the *K-value* in this paper.

$$N\propto M^{-b}$$ (12)

$$NT\_b\_value(i)=-\log_{10}(N-M+\max(N))$$ (13)

## 3 Results and discussion

Initially, the AE signals collected undergo analysis in the conventional time domain. The waveform distribution, cumulative event count, and time-load curve assist in approximating the location of the "collapse" of the AE signal system. Subsequently, the MED algorithm is applied to reduce noise, enabling further analysis of the AE signals in the natural time domain. By plotting eigenparameter maps, the critical state intervals of the AE signals are determined in the natural time domain. Additionally, the computation of the newly proposed NT_b_value yields K-values that provide an indication of the onset of the critical state.

### 3.1 Analysis of raw data in the traditional time domain

**Fig 5:**
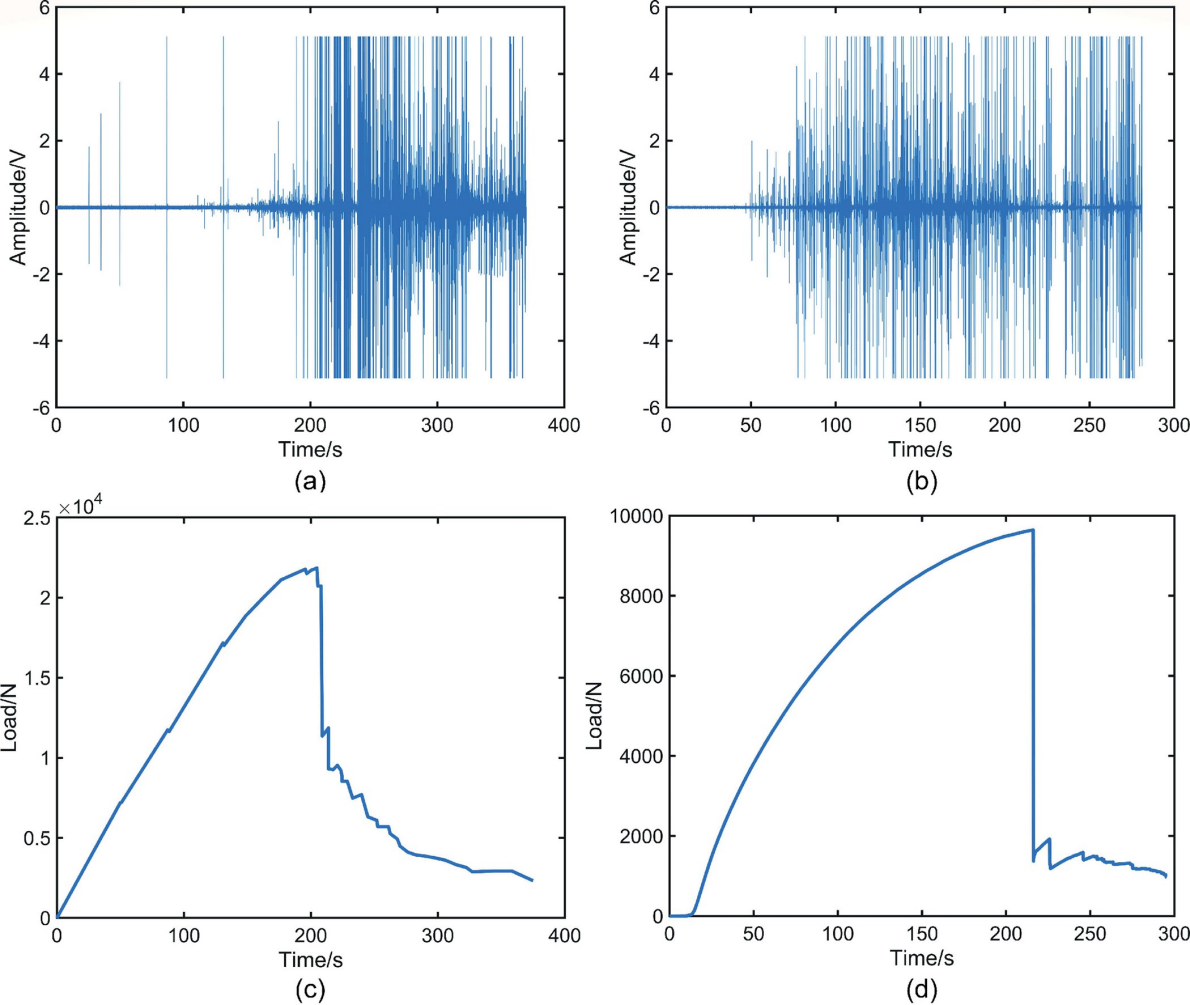
(a) Map of the AE signal of beech wood collected by the sensor. (b) Map of the AE signal of camphor pine collected by the sensor. (c) Time-loading diagram for beech wood during loading. (d) Time-loading diagram for camphor pine during loading.

The beech AE signal acquisition experiment lasted for 370 s, employing a sampling frequency of 500 kHz, resulting in a total of 1.85 × 10^8^ data signals, as illustrated in Fig 5(A). Fig 5(C) shows the time-load plot for the beech specimen, and it can be clearly seen that the pressurization value peaks at around 204 s, and at the same moment the AE signal in Fig 5(A) starts to appear intensive. This observation indicates that the beech specimen begins to fracture during this period, leading to the "collapse" of the beech AE system at around 204 s. Similarly, the Sphagnum’s AE signal acquisition experiment lasted for 281 s, employing a sampling frequency of 500 kHz, resulting in a total of 1.405 × 10^8^ data signals, as displayed in Fig 5(B). Fig 5(D) shows the time-loading plot of the sphagnum specimen, from which it can be observed that the pressurization reaches its maximum value near 216 s. However, the intensive appearance of the sphagnum AE signal in Fig 5(B) is not at this moment but near 85 s. In Fig 5(A) and 5(B), it is obvious that there is a big difference in the sustained fracture times obtained under the same experimental conditions, which is due to the fact that beech belongs to the family of arborvitae hardwoods and camphor pine belongs to the family of arborvitae softwoods. The difference is due to the different physical properties of the two. In summary, the characteristic value of the pressurization curve cannot be used as a reliable indicator to analyze the exact time of wood fracture. Analyzing AE waveforms in the conventional time domain only provides a basic understanding of the approximate time of the onset of wood fracture, but does not predict impending fracture. Therefore, analyzing AE waveforms in the conventional time domain has some limitations. The initial analysis in the traditional time domain can only provide an approximate estimation of the fracture initiation location in the wood, without the ability to further examine internal fractures or predict critical state intervals preceding fracture occurrence. Thus, it is necessary to transform the data into the natural time domain for detailed analysis.

### 3.2 MED

**Fig 6:**
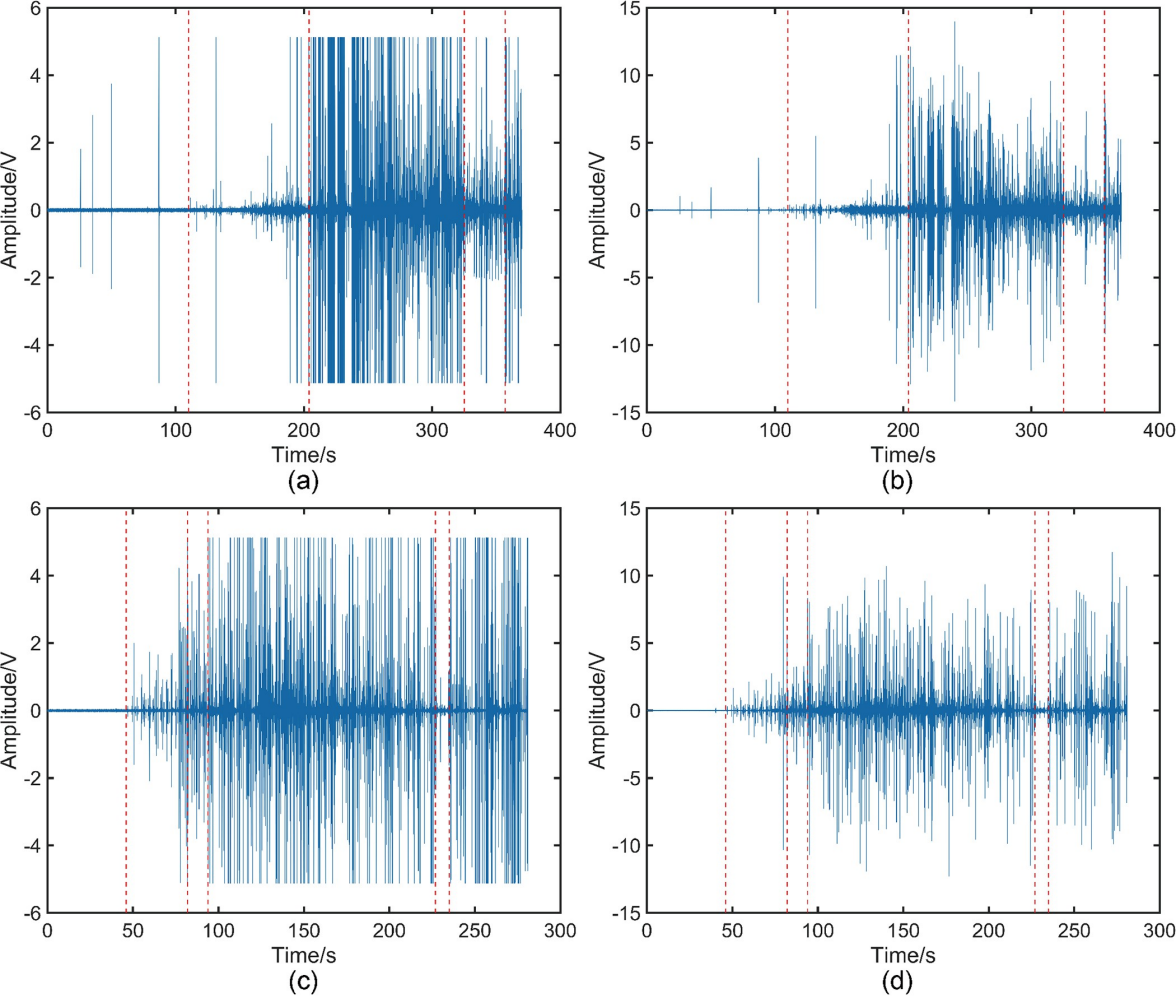
(a) Map of the AE signal of beech wood collected by the sensor. (b) AE signal map of beech wood after MED treatment. (c) Map of the AE signal of camphor pine collected by the sensor.(d) AE signal map of camphor pine after MED treatment.

**Fig 7:**
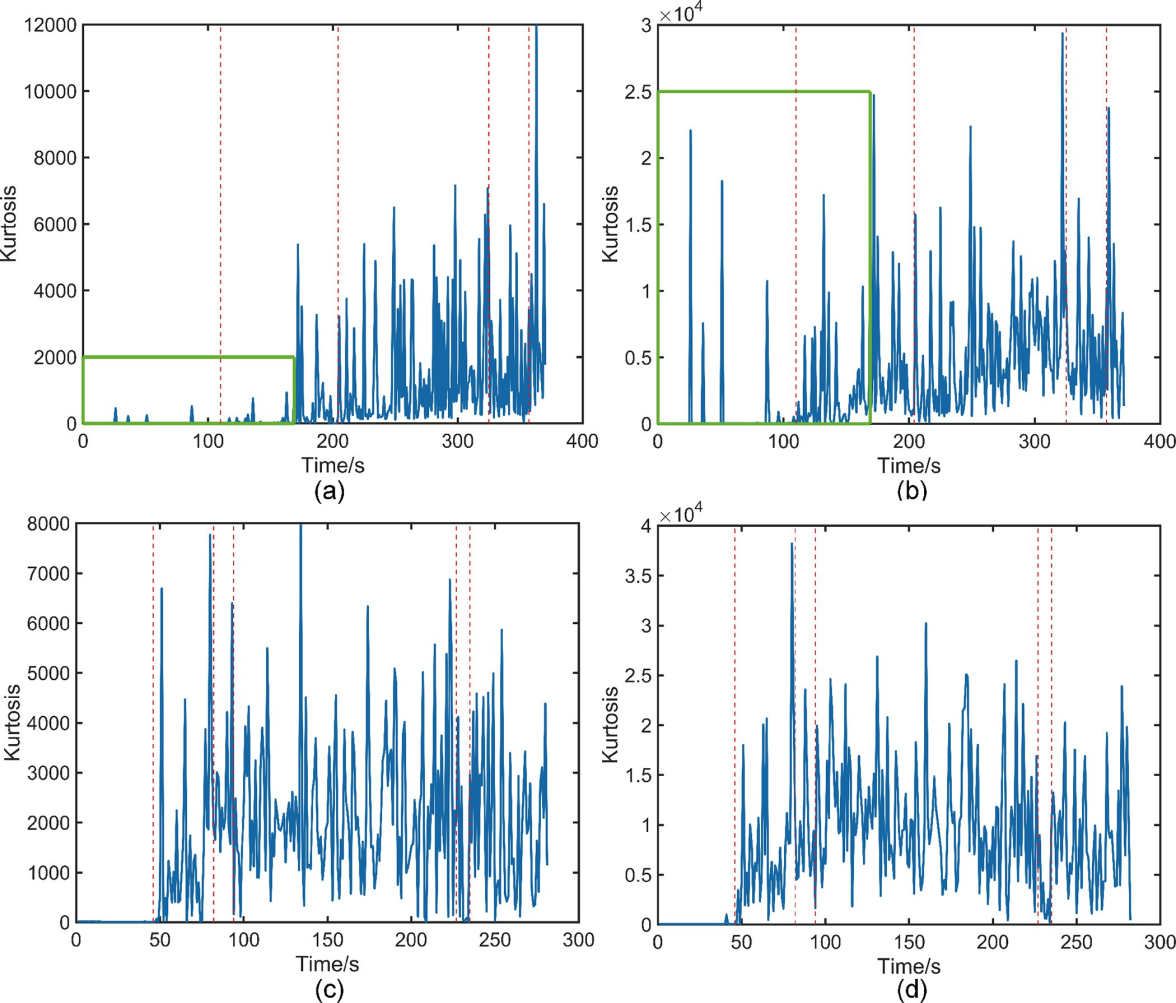
(a) Kurtosis curve of the AE signal of beech wood collected by the sensor. (b) Plot of the kurtosis value of the AE signal of beech wood after MED treatment. (c) Kurtosis curve of the AE signal of camphor pine collected by the sensor. (d) Plot of the kurtosis value of the AE signal of camphor pine after MED treatment.

Fig 6 depicts the waveform of the AE signal, with Fig 6(A) representing the raw AE signal of beech wood. In Fig 6(A), the signal can be roughly divided into five phases. The different phases are marked in the figure using red dotted lines. The first phase spans from 0 to 110 s, characterized by a limited number of AE signals with high amplitudes. The second phase encompasses 110 s to 204 s, during which the AE signal intensifies and its amplitude gradually increases, although the overall amplitude remains low. Moving to the third phase, from 204 s to 325 s, we observe the highest overall amplitude and the densest concentration of AE signals. The fourth phase spans 325 s to 357 s, marked by lower signal amplitudes compared to the third phase, yet still higher than the second phase. Finally, the fifth phase extends from 357 s until the end of the signal, with an increased signal amplitude compared to the previous phase, but overall amplitude similar to the second phase. Fig 6(B) displays the AE signal map obtained after applying the MED process to the original AE signal of beech wood. It is worth noting that it also shows five different phases, each of which is also labelled in the figure using red dashed lines, as can be clearly seen in Fig 6(B). The waveform distribution is very similar to Fig 6(A). The parameter kurtosis serves as an indicator of the transient amplitude distribution characteristics of vibration signals. In this experiment, the shock signal represents the effective signal, and a higher kurtosis value indicates a greater concentration of effective signals within a given time period. Fig 7(A) displays the kurtosis plot of the original AE signal obtained from beech wood, the phases have been marked using red dashed lines and the distribution of kurtosis values aligning with the AE signal distribution observed in Fig 6(A), effectively reflecting the AE signal distribution. Fig 7(B) presents the kurtosis values of the beech AE signals after undergoing MED processing. Comparing it with Fig 7(A), it is evident that the overall kurtosis values of the AE signals have significantly improved after MED processing, accentuating the impulse characteristics of the AE signals. For instance, the kurtosis values within the 0 s to 169 s interval exhibit relatively small values in Fig 7(A), and this area has been marked with a green box. However, in Fig 7(B), the kurtosis values in the intervals also marked using green boxes are significantly enhanced after MED treatment. Moving to Fig 6(C), it represents the raw AE signal obtained from camphor pine. The waveform can be roughly divided into six stages, which are labelled in the figure using the same red dotted line. The first stage spans from 0 s to 46 s, where virtually no valid AE signals are acquired. Transitioning to the second phase from 46 s to 82 s, AE signals begin to appear intensively with gradually increasing amplitudes, albeit overall low. The third phase encompasses the interval from 82 s to 94 s, displaying a decrease in amplitude instead of the expected increase observed in previous phases, resulting in an overall smaller amplitude compared to the second phase. The fourth stage extends from 94 s to 227 s, exhibiting the highest overall amplitude and a dense distribution of AE signals. Lastly, the fifth stage ranges from 227 s to 235 s. The amplitude of the signal in this stage is smaller than the amplitude in any of the stages, but the overall amplitude is still larger than the amplitude in the first stage. The sixth stage is from 235 s to the end of the signal, and the signal amplitude in this stage increases substantially compared to the previous stage, with the overall signal amplitude approaching that of the fourth stage. Fig 6(D) is a graph of the AE signal after MED processing from the original AE signal of camphor pine, in which it can still be roughly divided into six stages. The stages are separated using red dashed lines, and it can be seen that the waveform distribution of its stages is consistent with Fig 6(C). Fig 7(C) is the kurtosis map of the original AE signal of camphor pine, and after comparison, the distribution of the kurtosis value in the map is consistent with the distribution of the signal in Fig 6(C). Fig 7(D) showsthe kurtosis map of the AE signal kurtosis value of camphor pine after MED treatment. After comparing Fig 7(C) and 7(D) at various stages, it is found that the results are still consistent with the beech specimen, that is to say, the kurtosis values within the range of the effective AE signal are all enhanced.

Based on the aforementioned comparative analysis, it is evident that the overall trend of the AE signals remains unchanged following the implementation of the MED processing. However, the AE signals have undergone effective amplification and reduction, resulting in an overall waveform distribution that better aligns with the characteristics of AE signals. The observed enhancement in kurtosis values serves as compelling evidence that the MED algorithm successfully reduces noise in the AE signals acquired during this experiment. This achievement establishes a solid groundwork for subsequent analysis in the natural time domain.

### 3.3 Natural time domain analysis

The critical state intervals of the AE signals are identified in the natural time domain through the plotting of eigenparameters related to entropy (*S)*, inversion entropy (*RS)*, and variance (*κ*1). Subsequently, the critical state intervals identified in the natural time domain are converted into the conventional time domain. Additionally, the newly proposed *NT_b_value* calculation enables a detailed analysis of signal amplitude distribution, allowing for waveform analysis in segmented time intervals. To demonstrate the effectiveness of determining critical state intervals in complex AE signaling systems within the natural time domain, eigenparameter maps and *K-values* will be compared with "collapse" times determined using the conventional time domain. This comparison will showcase the efficacy of the natural time domain approach in determining critical state intervals and *K-values* for such systems.

#### 3.3.1 Characteristic parameter maps analysis

**Fig 8:**
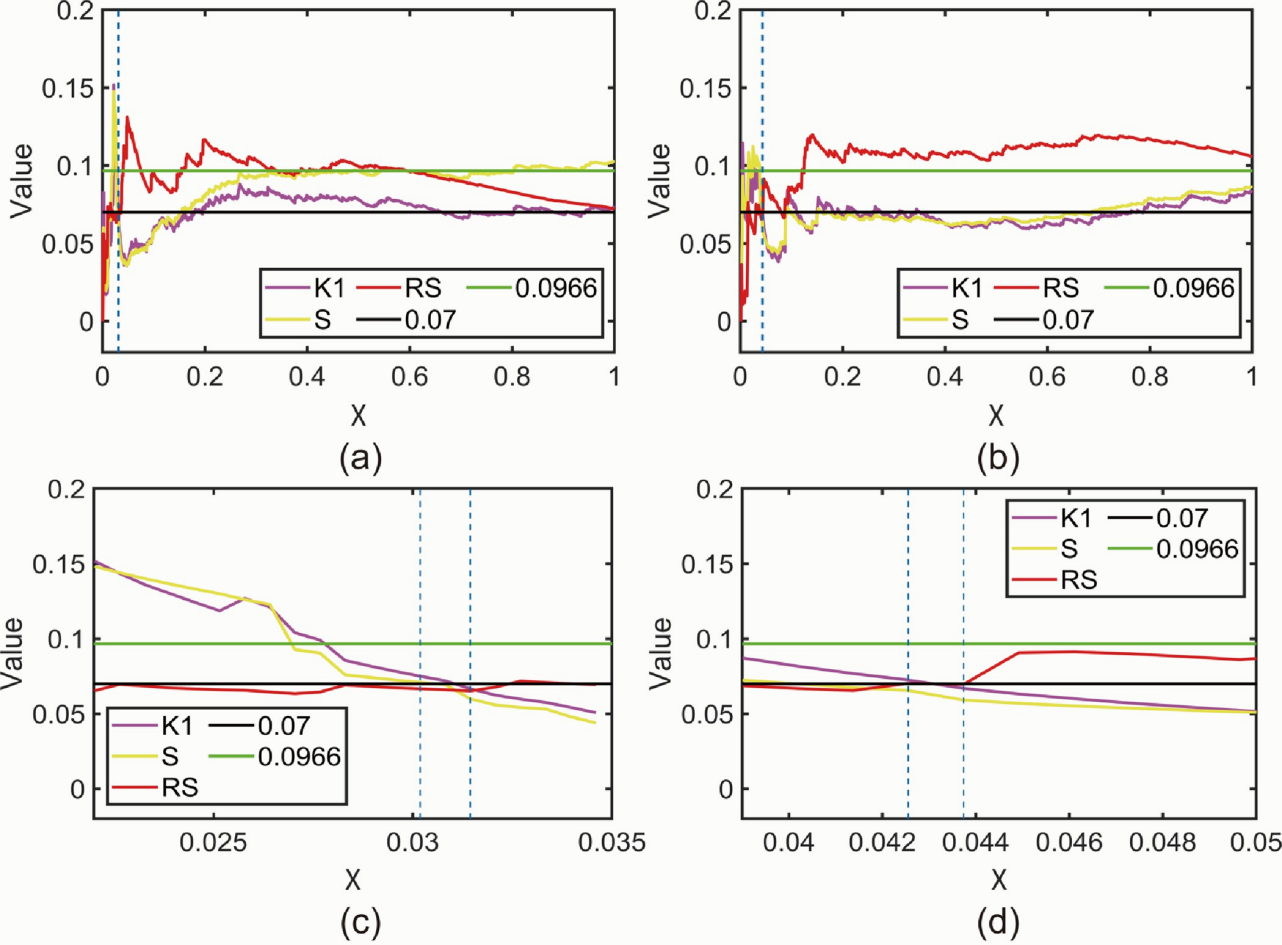
(a) Characteristic parameter maps of the MED-processed beech wood AE signal in the natural time domain. (b) Characteristic parameter maps of the MED-processed camphor pine AE signal in the natural time domain. (c) Local zoom-in of the characteristic parameters of the MED-treated beech wood AE signal in the natural time domain. (d) Local zoom-in of the characteristic parameters of the MED-treated camphor pine AE signal in the natural time domain.

Fig 8(A) shows the characteristic parameter maps of the AE signals of the beech specimen in the natural time domain after MED processing, with an effective number of events recorded as 1590. Similarly, Fig 8(B) shows the characteristic parameters of the AE signal of the MED-treated camphor pine specimen in the natural time domain, with an effective number of events recorded as 846. The intervals satisfying the critical state are identified within the Fig 8 using blue vertical lines, according to the conditions for satisfying the critical state intervals in section 2.4.1. However, due to the relatively small number of events within the critical state intervals compared to the overall number of events, the detailed information regarding these critical intervals is not clearly visible in Fig 8(A) and 8(B). Therefore, localized enlarged views of the critical intervals are shown in Fig 8(C) and 8(D) for the beechwood and camphor pine AE signals, respectively. In Fig 8(C), the interval in which the Beechwood AE signal meets the critical state condition in the natural time domain is identified as [0.0302, 0.0314], which corresponds to a time range of [195.99 s, 197.82 s] when converted to the conventional time domain. Similarly, in Fig 8(D), the interval in which the camphor pine AE signal meets the critical state condition in the natural time domain is identified as [0.0426, 0.0437], corresponding to a time range of [81.26 s, 81.87 s] in the conventional time domain. Based on the preliminary analysis using the conventional time domain, it is known that beech wood started to fracture around 204 s, whereas the range of critical intervals calculated in the natural time domain [195.99 s, 197.82 s] occurs earlier. Additionally, sphagnum began to fracture around 85 s, with the range of critical intervals calculated in the natural time domain [81.26 s, 81.87 s] also occurring earlier. Therefore, we can believe that compared with traditional time domain analysis, through natural time domain analysis of AE signals, it can be determined early that wood is about to enter a critical state. Table 1 provides a summary of information regarding AE signals from the beech specimen and the camphor pine specimen, as summarized according to Fig 8 The terms in Table 1 are explained as follows:

**Table 1:** Summary of information on beech and camphor pine AE signals.

	Beech	Camphor Pine
Event	1590	846
NT(event)	48–50	36–37
NT	0.0302–0.0314	0.0426–0.0437
t	195.99s-197.82 s	81.26 s-81.87 s

1. Event: Represents the number of events encompassed within each segment of the waveform in the natural time domain.
2. NT(event): Indicates the range of events corresponding to the critical state interval in the natural time domain.
3. NT: Denotes the critical state interval identified in the natural time domain, as indicated by the blue vertical line in Fig 8.
4. t: Represents the critical state interval in the conventional time domain.

**Fig 9:**
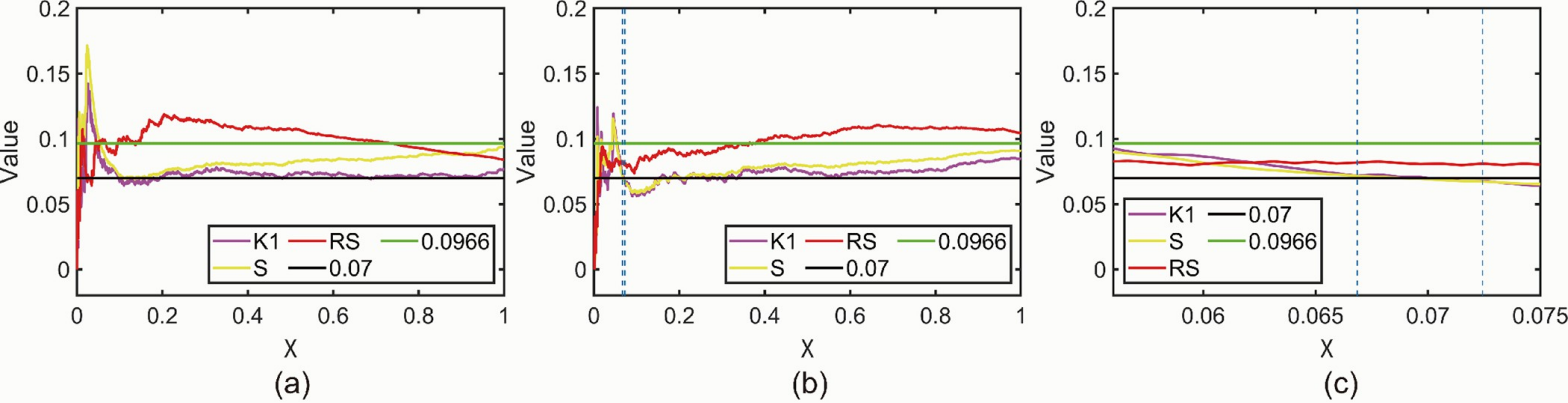
(a) Characteristic parameter maps of the original beech wood AE signal collected by the sensor in the natural time domain. (b) Characteristic parameter maps of the original camphor pine AE signal collected by the sensor in the natural time domain.(c) Local zoom-in of the characteristic parameters of the original camphor pine AE signal collected by the sensor in the natural time domain.

Fig 9 shows the characteristic parameter plot of the original AE signal in the natural time domain. Fig 9(A) shows the characteristic parameter plot of the original AE signal of the beech wood specimen in the natural time domain, and the effective number of events of the beech wood is 2793, but the range of intervals to satisfy the conditions of the critical state is not found in the plot. Fig 9(B) shows the characteristic parameter plots of the raw AE signals of the camphor pine specimen in the natural time domain, the effective number of events for beech is 1077, and the range of intervals that satisfy the critical state has been marked in the figure using blue vertical lines. Fig 9(C) is the local enlargement of the critical state interval of the original AE signal of the camphor pine specimen, in which it can be seen that the interval in which the original AE signal of the camphor pine specimen meets the conditions of the critical state in the natural time domain is [0.0669, 0.0724]. The range of the interval after converting it to the traditional time domain is [83.86 s, 85.95 s], and the beginning of the interval is earlier than 85 s, but the end of the interval is later than 85 s, and it cannot be used as the interval indicating the critical state. In summary, the MED-processed AE signals can effectively calculate the critical state interval range in the natural time domain, but the original AE signals not processed by MED cannot calculate the critical state interval range in the natural time domain. This reaffirms the excellent noise reduction capability of MED for the AE signals captured in this experiment.

#### 3.3.2 NT_b_value analysis

**Fig 10:**
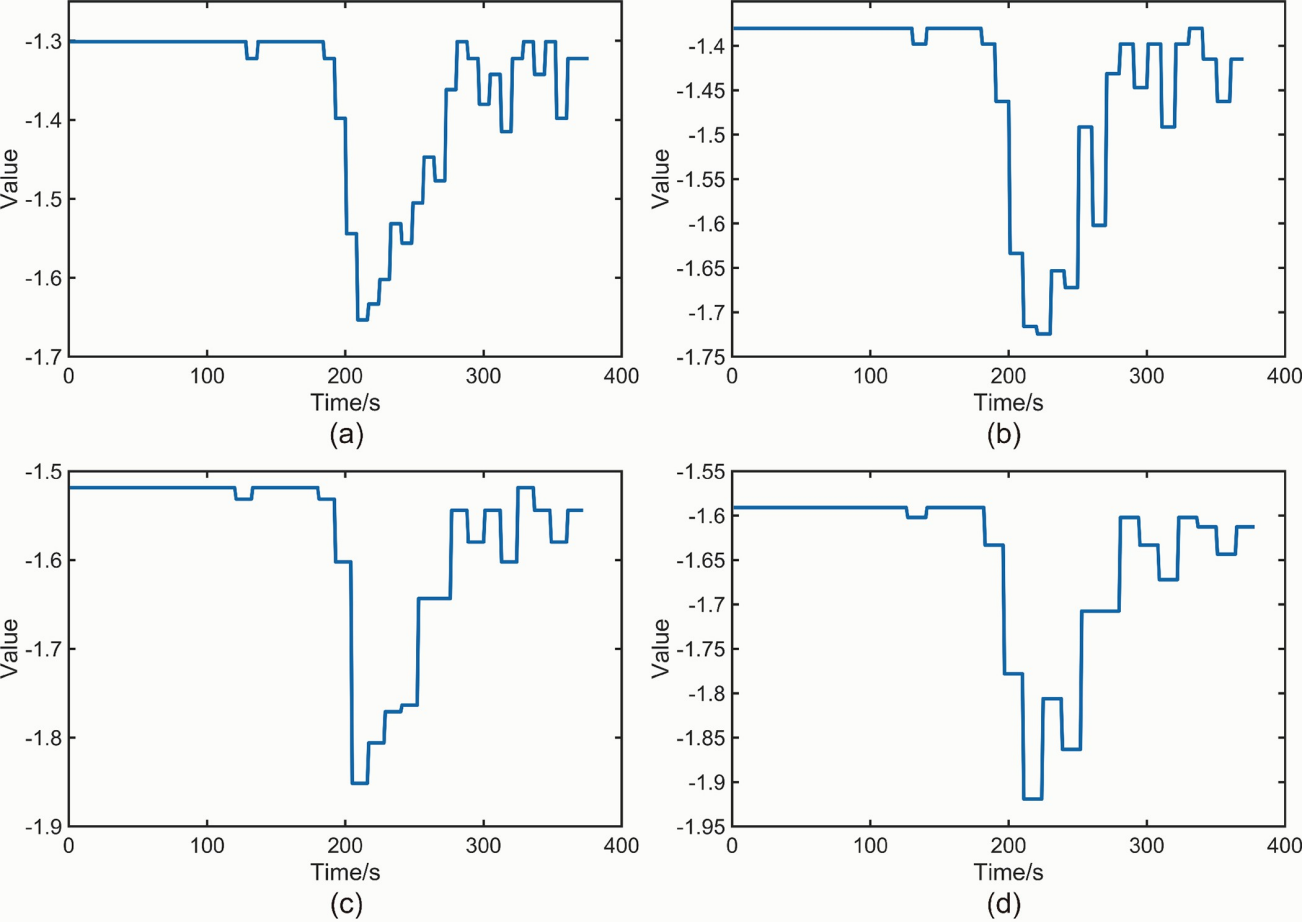
(a) *NT_b_value* plot for beech specimen *M* = 5,*t* = 8 s. (b) *NT_b_value* plot for beech specimen *M* = 5,*t* = 10 s. (c) *NT_b_value* plot for beech specimen *M* = 5,*t* = 12 s. (d) *NT_b_value* plot for beech specimen *M* = 5,*t* = 14 s.

Fig 10 shows the *NT_b_value* plots obtained for the beech specimen with the specified threshold *M* = 5. Fig 10(A)–10(D) correspond to the time intervals of 8 s、10 s、12 s and 14 s, respectively. Fig 10(A) can be analyzed according to the fluctuation of *NT_b_value* values in the following stages. The values in the interval 0 s-192 s are relatively smooth, generally fluctuating around the global maximum, indicating that the specimen has only experienced some small amplitude AE events within this interval and has not yet caused serious damage to the specimen. The value in the gap 193 s-200 s decreases from -1.322 in the previous interval to -1.398, and although the value decreases, the decrease is slight, so 193 s is not a *K-value* as defined in this paper. The value in the interval 201 s-208 s decreases from -1.398 to -1.544, which shows a substantial decrease compared to the previous period, indicating that the specimen starts to experience large amplitude AE events in this interval range, in other words, the *K-value* is 201 s. The values continued to decrease in the 209 s-216 s interval, reaching a global minimum, indicating that the most intensive large amplitude AE events occurred in the specimens within this interval. The values fluctuate in [-1.633,-1.301] from 217 s to 281 s, and the overall trend shows a slight increase, indicating that the number of small amplitude AE events is larger than the number of large amplitude AE events. However, large amplitude AE events still occur at the later stage of specimen fracture. The values fluctuated in [-1.301,-1.415] during the period after 281 s and up to the end of the period, and the overall mean value was close to the global maximum in the interval 0 s-192 s, indicating that the AE events at the end of the fracture of the specimen were predominantly small-amplitude events. In Fig 10(B), the values within the 0 s to 190 s interval generally fluctuate around the global maximum and remain relatively smooth. The values from 201 s to 210 s exhibit a significant decrease compared to the previous period, establishing 201 s as the *K-value*. Similarly, in Fig 10(C), the values within the 0 s to 192 s interval generally fluctuate around the global maximum. The values from 193 s to 204 s show a substantial decrease compared to the previous period, indicating a *K-value* of 193 s. Likewise, in Fig 10(D), the values within the 0 s to 182 s interval generally fluctuate around the global maximum and maintain smoothness. The values from 197 s to 210 s exhibit a substantial decrease compared to the previous period, establishing a *K-value* of 197 s. The fundamental trend observed in all four plots in Fig 10 remains consistent, although the specific values of K differ. Importantly, all *K-values* are earlier than the macroscopic fracture time of 204 s for beech wood.

**Fig 11:**
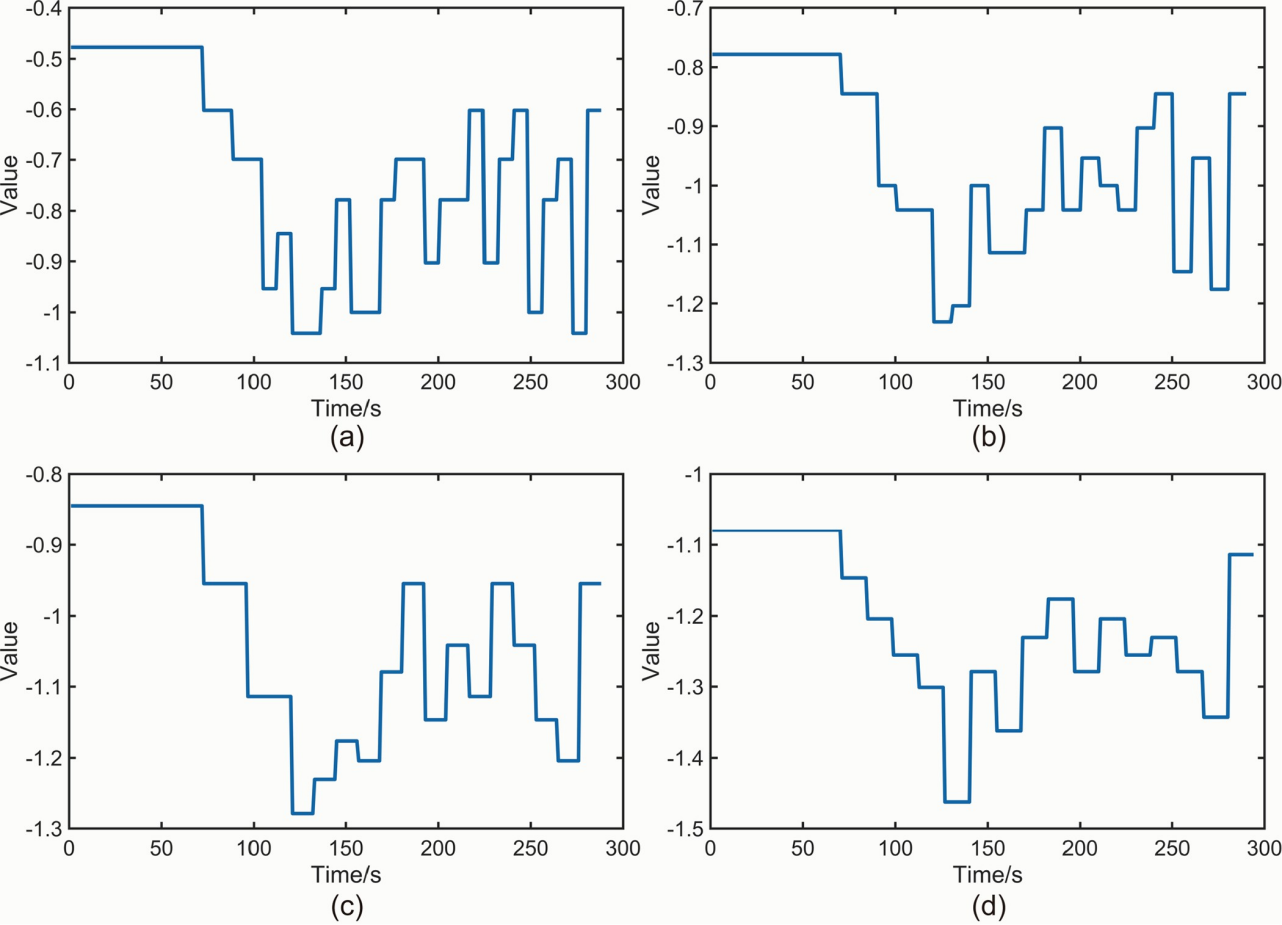
(a) *NT_b_value* plot of sphagnum pine specimen *M* = 5,*t* = 8 s. (b) *NT_b_value* plot of sphagnum pine specimen *M* = 5,*t* = 10 s. (c) *NT_b_value* plot of sphagnum pine specimen *M* = 5,*t* = 12 s. (d) *NT_b_value* plot of sphagnum pine specimen *M* = 5,*t* = 14 s.

Fig 11 illustrates the *NT_b_value* plot obtained for the camphor pine specimen using a specified threshold of *M* = 5. Fig 11(A)–11(D) correspond to time intervals of 8 s、10 s、12 s and 14 s, respectively. In Fig 11(A), the fluctuation of *NT_b_value* values allows for analysis in distinct stages. The values within the interval of 0 s to 72 s exhibit relative smoothness, generally fluctuating around the global maximum. This indicates that only a few small amplitude AE events have occurred in the specimen within this interval, without causing significant damage. The value from 73 s to 88 s decreases from -0.4771 in the previous interval to -0.6021, indicating the onset of a large amplitude AE event, establishing a *K-value* of 73 s for this interval. The values continue to decrease within the 89 s to 112 s interval, indicating an increased occurrence of large amplitude events during this phase. After 113 s, the value fluctuates within the range of [-1.041, -0.6021] with alternating ups and downs, indicating the simultaneous occurrence of both large-amplitude and small amplitude AE events in the late stage of Sphagnum rupture, with an equal number of these events. In Fig 11(B), the values within the 0 s to 70 s interval generally fluctuate around the global maximum and maintain smoothness. The values from 71 s to 80 s exhibit a substantial decrease compared to the previous period, establishing a *K-value* of 71 s. Similarly, in Fig 11(C), the values within the 0 s to 72 s interval generally fluctuate around the global maximum and remain relatively smooth. The values from 73 s to 84 s show a significant decrease compared to the previous period, indicating a *K-value* of 73 s. Likewise, in Fig 11(D), the values within the 0 s to 70 s interval generally fluctuate around the global maximum and maintain smoothness. The values from 71 s to 84 s exhibit a significant decrease compared to the previous period, establishing a *K-value* of 71 s. The fundamental trend observed in all four plots in Fig 11 remains consistent, although the specific values of K differ. Importantly, all *K-values* occur earlier than the macroscopic fracture time of 85 s for the sphagnum pine specimen.

**Fig 12:**
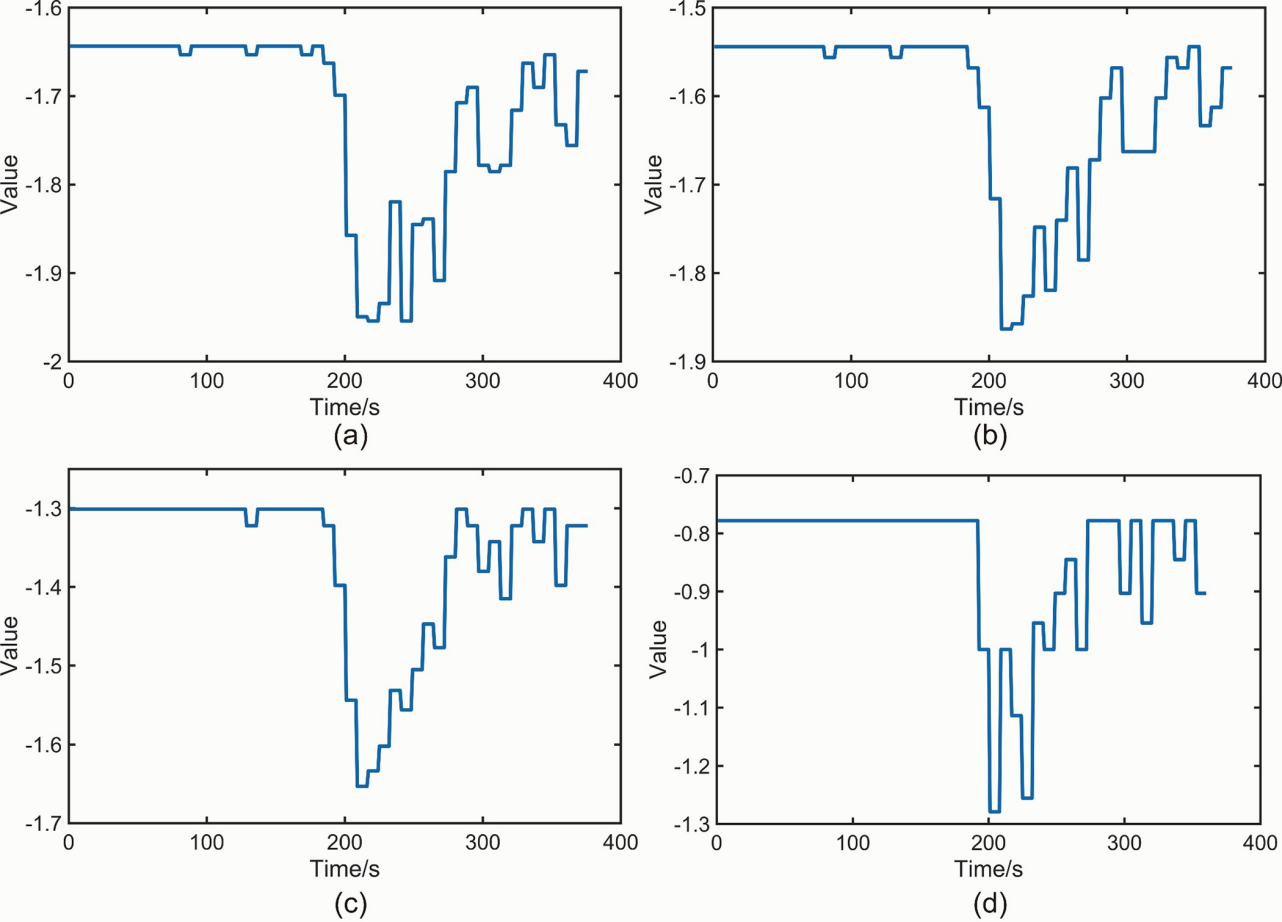
(a) *NT_b_value* plot for beech specimen *M* = 2,*t* = 8 s. (b) *NT_b_value* plot for beech specimen *M* = 3,*t* = 8 s. (c) *NT_b_value* plot for beech specimen *M* = 5,*t* = 8 s. (d) *NT_b_value* plot for beech specimen *M* = 7,*t* = 8 s.

**Fig 13:**
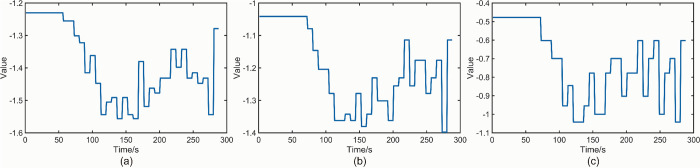
(a) *NT_b_value* plot of sphagnum pine specimen *M* = 2,*t* = 8 s. (b) *NT_b_value* plot of sphagnum pine specimen *M* = 3,*t* = 8 s. (c) *NT_b_value* plot of sphagnum pine specimen *M* = 5,*t* = 8 s.

Table 2 presents a summary of the *K-values* obtained from Figs 10 and 11. It is observed that the effect on *K-values* is minimal when the M-value is fixed and different values of t are considered. The value of *t* is flexible and can be chosen according to specific requirements. Fig 12 displays the *NT_b_value* plot for the beech specimen within the specified time interval of *t* = 8 s. Fig 12(A)–12(D) correspond to *M* values of 2、3、5 and 7, respectively. Similarly, Fig 13 illustrates the *NT_b_value* plot for the sphagnum pine specimen within the specified time interval of *t* = 8 s. Fig 13(A)–13(C) correspond to *M* values of 2、3 and 5, respectively. Table 3 provides a summary of the *K-values* obtained from Figs 12 and 13. Once again, the impact on the *K-value* remains relatively small when the *t* value is fixed and *M* takes different values. However, it is important to note that excessively large *M* values can render the *NT_b_value* meaningless. *NT_b_value* demonstrates robustness to changes in *M* and *t* values, but it is crucial to analyze different wood species using specific approaches. The recommended parameter combinations for beech are *M* < 5 and *t* < 10, while for camphor pine, it is advisable to use *M* < 5 and *t* < 14.

**Table 2:** Summary of *K-values* for Figs 10 and 11.

	t = 8 s	t = 10 s	t = 12 s	t = 14 s
K-value of beech	201 s	201 s	193 s	197 s
K-value of sphagnum pine	73 s	71 s	73 s	71 s

**Table 3:** Summary of *K-values* for Figs 12 and 13.

	M = 2	M = 3	M = 5	M = 7
K-value of beech	201 s	201 s	201 s	193 s
K-value of sphagnum pine	73 s	81 s	73 s	pointless

In summary, the characteristic parameter analysis conducted in the natural time domain reveals that the critical state time range for beech trees is [195.99 s, 197.82 s]. Furthermore, the *NT_b_value* analysis in the natural time domain yields a *K-value* of 201 s. Both of these values indicate a time earlier than 204 s compared to the "collapse" time of the AE system analyzed in the conventional time domain. Similarly, for sphagnum pine, the characteristic parameter analysis in the natural time domain determines a critical state time range of [81.26 s, 81.87 s]. The *NT_b_value* analysis in the natural time domain yields a *K-value* of 81 s. Notably, both of these values are relatively close and precede the AE "collapse" time of 85 s observed in the conventional time domain. Therefore, it is evident that the critical time preceding the "collapse" of the AE system can be effectively determined through either the analysis of characteristic parameters in the natural time domain or the *NT_b_value* analysis for both camphor pine and beech.

## 4 Conclusion

Wood is a widely used material in ancient architecture and it is important to monitor its health. Our group has previously addressed the difficulty of distinguishing between microcracked AE signals and deformed AE signals in the classification model of AE signals in wood. This is due to the fact that the critical state in the wood fracture process is just in the interval of microcracked AE signals, and the classification model established in the previous stage does not take into account the complexity of the critical state AE signals, and lacks the classification features that differentiate the microcracked acoustic emission signals from other kinds of AE signals. The feature parameters in the natural time domain proposed in this study can be used as features to distinguish microcracked AE signals from deformed AE signals, which in turn improves the classification accuracy of the 2 AE signals. In this study, the AE signals during wood fracture were collected by three-point bending load experiments, and the AE signals were converted to the natural time domain, which is different from the traditional time domain analysis, for the characteristic parameter analysis. On the one hand, the characteristic parameters S, RS and *κ*1 are used to determine the critical state time interval during wood fracture; on the other hand, the "collapse" time of the AE system is determined by the *K-value* obtained from the analysis of the characteristic parameter *NT_b_value*. The results show that the analysis in the natural domain outperforms the analysis in the traditional time domain, and the characteristic parameters in the natural time domain can be captured before the AE system undergoes a "collapse" event, thus determining the critical state interval of the AE system. In addition, the analysis of *NT_b_value* in the natural time domain can obtain the amplitude distribution of AE events in each customized time interval, which further provides more detailed characterization information for health monitoring of wood structures. Table 4 summarises the important information in this study. In this study, the predicted critical state time intervals in the natural time domain for both beech and camphor pine samples are earlier than the time when the samples undergo "collapse" in the conventional time domain. Thus the AE signal analysis method based on the natural time domain proposed in this paper is effective in predicting the arrival of the critical state. This method provides a new idea for health monitoring of wooden building components, which can convert the real-time monitored AE signals to the natural time domain and obtain the features that distinguish them from the traditional time domain for further analysis, so as to determine the health status of wooden components. Considering that wood is a complex anisotropic material, the next step could be to use multi-angle, high-precision sensors for data acquisition in order to study the interconnection of multi-channel data in the spatial domain.

**Table 4:** Summary of important information.

	Beech	Camphor Pine
**Critical state interval in conventional time**	**195.99 s-197.82 s**	**81.26 s-81.87 s**
**Critical state intervals in natural time**	**0.0302–0.0314**	**0.0426–0.0437**
**"Collapse" time of the AE system**	**204 s**	**85 s**
**K-value (M = 5, t = 8 s)**	**201 s**	**73 s**
**Load conditions**	**Constant displacement pressurisation**	

## Supporting information

S1 Text Master function for plotting parameters in the natural time domain. (TXT)

S2 Text Subfunction to compute the improved b_value. (TXT)

S3 Text Subfunctions for calculating variance. (TXT)

S4 Text Calculate the subfunctions of the normalised energy. (TXT)

S5 Text Calculate the subfunctions of the inverse entropy. (TXT)

S6 Text Calculating subfunctions of entropy. (TXT)

S7 Text Compute subfunctions for natural time events. (TXT)
